# Physicochemical and Biological Characterization of rhC1INH Expressed in CHO Cells

**DOI:** 10.3390/ph14111180

**Published:** 2021-11-19

**Authors:** Ekaterina Zubareva, Maksim Degterev, Alexander Kazarov, Maria Zhiliaeva, Ksenia Ulyanova, Vladimir Simonov, Ivan Lyagoskin, Maksim Smolov, Madina Iskakova, Anna Azarova, Rahim Shukurov

**Affiliations:** JSC “GENERIUM”, 14, Vladimirskaya Street, Volginskiy 601125, Vladimir Region, Russia; degterev@ibcgenerium.ru (M.D.); kazarov@ibcgenerium.ru (A.K.); zhiliaeva@ibcgenerium.ru (M.Z.); ulyanova@ibcgenerium.ru (K.U.); simonov@ibcgenerium.ru (V.S.); lyagoskin@ibcgenerium.ru (I.L.); smolov@ibcgenerium.ru (M.S.); iskakova@ibcgenerium.ru (M.I.)

**Keywords:** rhC1INH, C1 inhibitor, Berinert^®^, Ruconest^®^, analytical methods, characterization, CHO cells, hereditary angioedema (HAE), acquired angioedema (AAE)

## Abstract

The disfunction or deficiency of the C1 esterase inhibitor (C1INH) is associated with hereditary or acquired angioedema (HAE/AAE), a rare life-threatening condition characterized by swelling in the skin, respiratory and gastrointestinal tracts. The current treatment options may carry the risks of either viral infection (plasma-derived Berinert^®^) or immune reaction (human recombinant C1INH from rabbit milk, Ruconest^®^). This study describes the physicochemical and biological characterization of a novel recombinant human C1 esterase inhibitor (rhC1INH) from Chinese hamster ovary (CHO) cells for the treatment of hereditary angioedema compared to the marketed products Berinert^®^ and Ruconest^®^. The mass spectrometry results of total deglycosylated rhC1INH revealed a protein with a molecular mass of 52,846 Da. Almost full sequence coverage (98.6%) by nanoLC-MS/MS peptide mapping was achieved. The purity and C1s inhibitory activity of rhC1INH from CHO cells are comparable with Ruconest^®^, although we found differences in charge isoforms distribution, intact mass values, and N-glycans profile. Comparison of the specific activity (IC_50_ value) of the rhC1INH with human C1 esterase inhibitor from blood serum showed similar inhibitory properties. These data allow us to conclude that the novel rhC1INH molecule could become a potential therapeutic option for patients with HAE/AAE.

## 1. Introduction

Hereditary angioedema (HAE) is a rare disease having an orphan status. The study of HAE began in 1962 by Landerman and colleagues [1]. The main manifestations of angioneurotic edema are uncontrolled swelling over the body due to either decreased amounts or dysfunctional C1 inhibitor protein (type I HAE and type II HAE, respectively) [2].

The biological role of C1 esterase inhibitor (C1 inhibitor) is to regulate two mutually dependent processes by inhibition of serine proteases responsible for vascular permeability and inflammatory response. Under normal circumstances, the C1 inhibitor regulates the contact system, also known as the kallikrein-kinin system. The activation of this system results in bradykinin generation. Contact system regulation occurs due to inhibition of the first complement component of the C1 macromolecule. The anti-inflammatory effect of the C1 inhibitor is exerted via regulation of the complement system activation. A recent study demonstrated that the C1 inhibitor inactivates both C1r and C1s [3]. The C1 inhibitor also regulates the lectin pathway via the inactivation of MASP1 and MASP2 [4,5].

Recent studies defined a connection between the fibrinolytic system and the complement system in the HAE pathogenesis. The fibrinolytic system is associated with bradykinin-forming cascade at several levels. The interaction between the fibrinolytic system and the contact system leads to an auto-amplification loop that sustains the generation of bradykinin, the main mediator of HAE attacks. At the same time, the classical complement pathway can be activated by plasmin. As a result of this process, the mediators of inflammation are released, such as plasmin, anaphylatoxins (especially C3a and C5a) and histamine. The development of HAE is also associated with allergic diseases [6].

The plasminogen activation system is mainly known for its function as a key component of the fibrinolytic cascade and is crucial in the maintenance of balance between coagulation and fibrinolysis. The knowledge of how a complement system is involved in the generation of immunologic and/or allergic diseases clarifies some fundamental molecular aspects of the link between the fibrinolytic system and the complement system in the HAE pathogenesis [6].

Factor XII, plasma prekallikrein, factor XI and high molecular weight protein kininogen are the major components of the plasma kallikrein-kinin system (contact system). Deficiency in the functional C1 inhibitor results in the unregulated activation of factor XII that initiates kallikrein formation. The newly formed complex cleaves high molecular weight kininogen, leading to the generation of excessive free bradykinin, a potent vasoactive peptide resulting in edema [7,8].

The C1 esterase inhibitor is a heavily glycosylated single chain polypeptide protein. It consists of 365 amino acids including a C-terminal serpin domain and a unique nonserpin N-terminal domain of 113 amino acids. The amino acid sequence of the serpin domain shows clear homology to other serpins and plays a main role in the biological activity of the molecule. Two disulfide bonds are formed between cysteine residues (Cys^101^ and Cys^108^) at the N-terminus of the nonserpin domain and two cysteines (Cys^406^ and Cys^183^) at the serpin domain, respectively. These two disulfide bridges stabilize the metastable conformation of the central β-sheet, which is responsible for serpin activities [9]. The N-terminal domain of the C1 inhibitor mainly contains O- and N-linked glycosylation sites [10]. Over the past five years, several C1 inhibitor replacement therapies for the treatment and prophylaxis of HAE have been marketed in the United States and Europe, including C1 inhibitors from pooled human blood plasma (plasma-derived human C1 inhibitors, pd-hC1INH; Berinert^®^, Haegarda^®^ and Cinryze^®^), as well as recombinant protein from transgenic rabbits (conestat alfa, Ruconest^®^). However, plasma-derived C1 inhibitors have some safety drawbacks. The protein manufactured by recombinant DNA technology is preferable because manufacturing the rhC1INH does not depend on blood supply and has no risk of viral contamination. Although Ruconest^®^ has the same amino acid sequence as pd-hC1INH, it is purified from the milk of transgenic rabbits; thus, its glycosylation profile is different from that of human plasma-derived C1 inhibitor. As a result, Ruconest^®^ has an extremely short half-life of about 2.4–2.7 h [11]. Also, product release depends on the age of animals, amount of milk, animal’s health condition, etc. Conestat alfa also carries a risk of allergic reactions due to the presence of potential immunogenic impurities. Attempts to produce the C1 inhibitor in bacterial and yeast systems did not give rise to any commercially viable products [9,11,12]. This may be explained by the low expression level of the target protein, its inactivation and/or non-mammalian glycosylation profile that makes it unsuitable for clinical use. However, mammalian cell systems make it possible to manufacture a molecule with a clinically suitable structure and activity profile.

Here we report the results of the physicochemical and biological characterization of the novel rhC1INH manufactured by recombinant DNA technology in the CHO cell line in comparison to two commercially available C1 esterase inhibitors: Ruconest^®^ and Berinert^®^.

To assess physicochemical and biological properties of the novel rhC1INH expressed in the genetically engineered Chinese hamster ovary (CHO) cells, the state-of-the-art robust approaches, including chromatographic, electrophoretic and bioassay methods, were employed. The selected methods can detect the differences in the protein structure, as well as identify and quantify protein variants and assess critical quality attributes of the molecule [13].

Our results show that the chemical and biological properties of the rhC1INH from CHO cells allow it to potentially be used to effectively treat various complement-mediated disorders. The manufacturing process of rhC1INH from the CHO cell line is easily controllable and manageable at the cultivation stage than the protein isolated from blood or rabbit milk and allows to obtain the product with the expected properties.

## 2. Results

### 2.1. Obligatory Quality Attributes of the Novel rhC1INH

The newly manufactured recombinant human C1 esterase inhibitor (rhC1INH) is a recombinant analogue of human C1INH that is purified from the genetically engineered Chinese hamster ovary (CHO) cell line. The molar extinction coefficient of the rhC1INH is determined as 27,851 ± 208 M^−1^ cm^−1^, which corresponds to the absorption value of a 0.1% solution $E_{280}^{0.1\%}$ = 0.53. The osmolality of the solution is 440 ± 45 mOsm/kg; pH 7.1 ± 0.1.

### 2.2. MolecularMass Determination by LC-ESI-MS

An estimated average molecular mass of the rhC1INH is determined by its amino acid composition after its total deglycosylation. Detection of the total mass of intact rhC1INH by LC-MS is hampered by its heavy glycosylation. The number of C1 inhibitor proteoforms is too large to achieve well-resolved mass spectra in native mass spectrometry.

**Figure 1:**
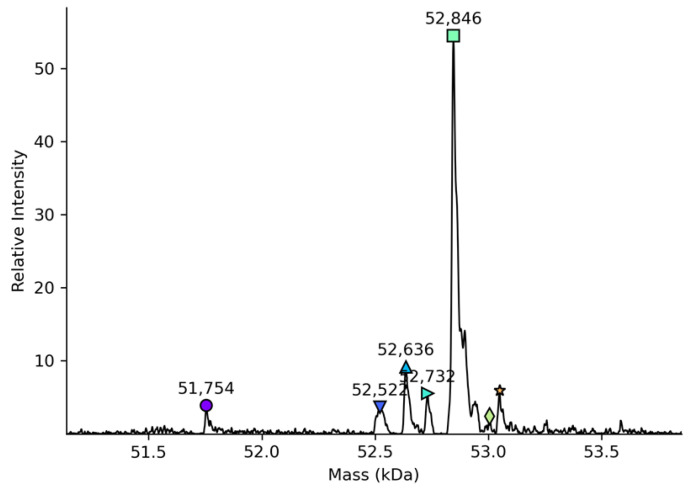
Mass-spectrometry data of the total deglycosylated variants of rhC1INH mass distribution. The integrated peaks are designated by colorful symbols of different shapes.

The average molecular mass of the totally deglycosylated rhC1INH molecule is about 52,846 Da which is similar to the theoretical value (52,849 Da) obtained from Expasy/ProtParam tool [14] (Table 1, Figure 1). The molecular mass was calculated based on the presence of six total deamidation sites after the PNGase F N-glycosylation treatment and oligosaccharides elimination.

The data in Figure 1 show the presence of a degradation product, which appears to be a truncated molecule, lacking several N-terminus amino acids. We also found a minor amount of protein with incomplete deglycosylation (since 53.0 kDa, Figure 1). Information on the molecular weight of a fully deglycosylated molecule confirms its authenticity by matching the expected and measured average molecular weights.

### 2.3. Amino Acid Sequence Validation and Peptide Mapping

Peptide mapping allows the general comparison of the primary (amino acid) sequence of proteins by testing the set of peptides formed as a result of site-specific proteolysis. This test is sensitive enough to detect differences between closely related proteins.

**Figure 2:**
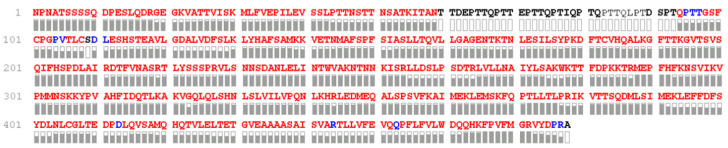
Map of the rhC1INH primary structure. The identified amino acids are highlighted with bold; red—amino acids with both b- and y-ions; blue—amino acids with one b- or y-ion; black—amino acids with no individual fragment ions (the last are located preferably at the hyperglycosylated part). Gray rectangles are a graphical representation of the reliability of the identification of a particular amino acid residue, including the number of fragment ions and their intensities.

Because of the high complexity of the rhC1INH molecule, its amino acid sequence was validated using a bottom-up proteomic approach with multi-enzyme treatment and nanoLC-MS/MS analysis of resulting peptides prior to peptide mapping. The protocol allows achieving almost full sequence coverage (98.6%) (Figure 2).

**Figure 3:**
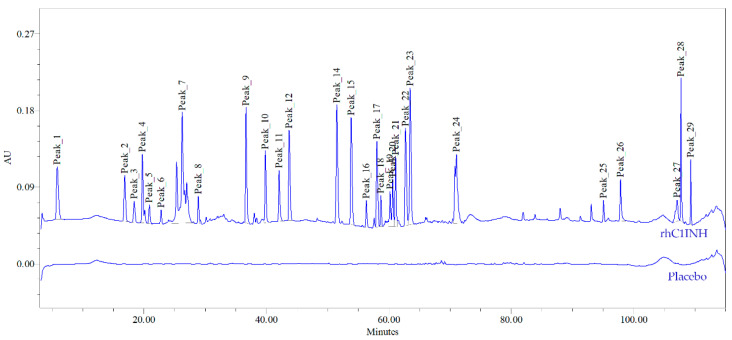
The rhC1INH peptide mapping chromatogram.

Next, we performed the peptide mapping assisted by trypsin proteolysis and LC-UV-MS/MS analysis with an analytical HPLC flow range. Chromatographic profiles of rhC1INH tryptic digests consisted of more than 29 peptide peaks that match 80% of the rhC1INH amino acid sequence (Figure 3, Table S1). The total number of peptides is difficult to define due to the heavy glycosylation of the C1 inhibitor [15].

### 2.4. High-Order Structure

Serpins inhibit serine proteases by forming irreversible equimolar complexes. In addition, serpin proteases lose their activity after interaction with the substrate, so they are often called “suicidal” proteins. The active center of C1INH is located on the flexible reactive loop, which can interact with target proteases. In the active form the reactive center loop protrudes from the bulk of the protein and presents the P1–P10 residues as a substrate for the proteolytic attack [16]. Upon binding the target protease, the reactive center loop inserts itself into the serpin’s central β-sheet, while the attached protease moves to the opposite pole of the serpin disrupting the protease’s active site [17]. This principle of inhibition can be realized if a high-order structure has a correct conformation.

The secondary structure (α-helixes, β-sheets, and “random” structures) and the tertiary structure of the C1 inhibitor is determined by circular dichroism (CD) spectroscopy in the far UV region (Figure S1a) and near UV region (Figure S1b), respectively. The rhC1INH is characterized by approximately equal contributions of α-helices (29.4–30.1%), and disordered structure (29.3–29.5%). The study of the secondary structure by Fourier transform infrared spectroscopy (FTIR) (Figure S1c) showed that the rhC1INH protein is characterized by an almost equal contribution of the β-sheet structure (about 29.9%), α-helices (about 29.7%) and disordered structure (about 31.4%), which is in good agreement with the results obtained by the CD method.

The rhC1INH contains three tryptophan residues and seven tyrosine residues that enabled us to use the protein fluorescence to study the state of the local environment of amino acid residues in this molecule. The fluorescence parameters of tryptophan residues, in contrast to tyrosine and phenylalanine residues, are very sensitive to the microenvironment [18]. The shape of the fluorescence spectra (Figure S1d) indicates the predominance of the tryptophan residues spectra. The maximum fluorescence spectrum position at 327.6 nm (λ− = 306.6 nm; λ+ = 356.9 nm) indicates a relatively low availability of tryptophan residues to the solvent, which provides the predominant spectral contribution. The novel rhC1INH belongs to Class I proteins according to classification based on tryptophan residue type. This classification is based on the correlation between the spectral and structural classes of tryptophan residues. This type of molecule can form the exciplexes with 2:1 stoichiometry. rhC1INH protein has a lower packing density, which could lead to greater mobility of the environment than other classes of proteins [18].

The quaternary structure of the rhC1INH was determined by dynamic light scattering. A single light scattering peak with a mass fraction close to 100% was observed. The hydrodynamic diameter was about 9.8 nm (Figure S2). 

The thermodynamic stability of the rhC1INH was evaluated by differential scanning calorimetry (DSC). The DSC thermograms of the rhC1INH are found to be superimposable. The samples show a single pronounced thermal denaturation peak with a maximum at around 57 °C (Figure S2).

Free thiols content analysis of the rhC1INH molecule showed the absence of available sulfhydryl groups in the protein denatured under non-reducing conditions (average is 0.47 ± 0.01 units). The analysis of thiols carried out after the reduction in disulfide bonds with dithiothreitol established the presence of about four sulfhydryl groups in the protein structure (average is 3.94 ± 0.21 units), which corresponds to the content of two disulfide bonds in the protein structure and agrees with the theoretically expected result and literature on the Berinert^®^ structure [19].

### 2.5. Size Heterogeneity

#### 2.5.1. Electrophoretic Analysis of the Molecular Weight

**Figure 4:**
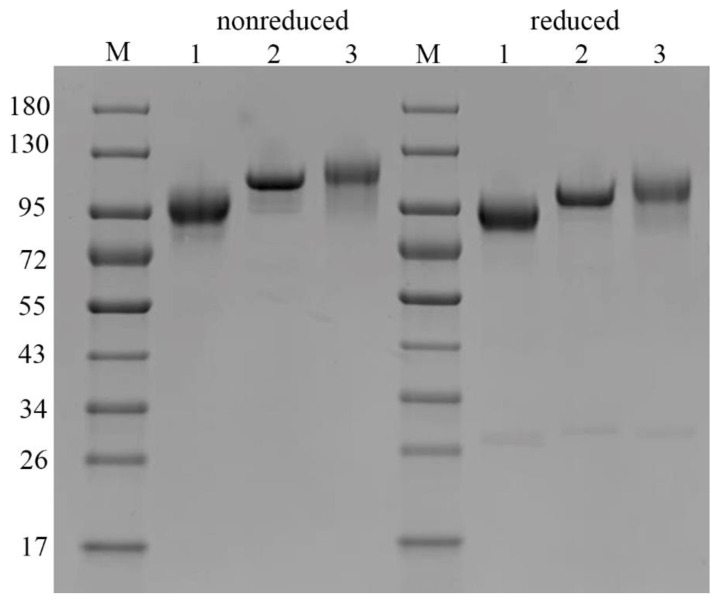
Electropherogram of the various C1 inhibitor molecules under non-reduced (on the left) and reduced (on the right) conditions. #1—Ruconest^®^, #2—Berinert^®^, #3—rhC1INH, M—molecular weight markers (kDa).

Comparison of the electrophoretic profiles of proteins by sodium dodecyl sulfate polyacrylamide gel electrophoresis (SDS-PAGE) under reducing and non-reducing conditions can be useful to learn about the nature of covalent bonds. Noncovalent aggregates are usually not detected because SDS disturbs interactions alike [20]. Thus, the SDS-PAGE technique can assess the weight of monomers and fragments (Figure 4).

One major band was observed for all samples, both under reducing and non-reducing conditions. The broadening of these bands was caused by the heterogeneity of glycosylation pattern, which is reviewed later in this article (part 2.8. “Glycosylation”). The additional impurity bands of fragments (30 ± 1 kDa) were also found (Table 2).

#### 2.5.2. Size Exclusion Chromatography

**Figure 5:**
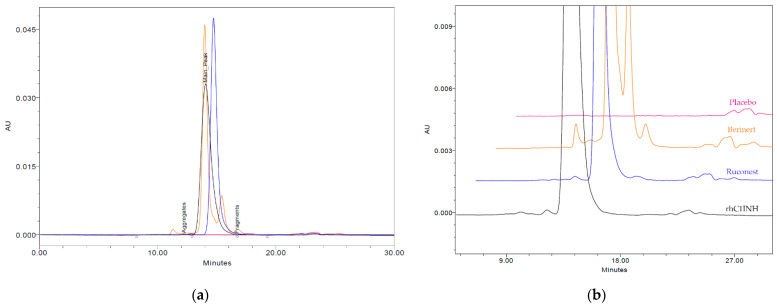
Comparison of size-exclusion chromatograms of the rhC1INH (black line), Berinert^®^ (yellow line), Ruconest^®^ (blue line) and placebo buffer (red line); (**a**) full view of the chromatogram, (**b**) enlarged view of impurities peaks.

Since protein aggregates of the biologic immunomodulators are known to trigger an unwanted immunogenic response [21], we performed size exclusion chromatography (SEC) to detect the levels of aggregates, monomers and fragments in the rhC1INH formulation. The retention times of the monomer of the rhC1INH, plasma-derived Berinert^®^ and recombinant Ruconest^®^ vary because of the differences in glycan profile (part 2.8. «Glycosylation»), which is consistent with the literature [22]. The monomer purity for the rhC1INH was 98,71 ± 0.23%, 98.96 ± 0.05% for Ruconest^®^ and 74.05 ± 0.31% for Berinert^®^ (Table 3 and Figure 5).

SEC revealed the lowest amount of protein fragments and low molecular weight (LMW) impurities for the novel rhC1INH compared to both commercial products. The amount of aggregates was similar to Ruconest^®^ but significantly lower than that of Berinert^®^. While the main peak in Berinert^®^ and in rhC1INH had an elution time of about 14.0 min, for Ruconest^®^ active pharmaceutical ingredient (API) there was a shift in elution time close to 15.0 min (Figure 5). The relative standard deviation (%RSD) of retention time for the main peak did not exceed 3.86%. The height of the main peak of the rhC1INH was less than for Berinert^®^ and Ruconest^®^ API due to the broader main peak in the rhC1INH despite the same amount of protein injected.

### 2.6. Reverse-Phase Chromatography

**Figure 6:**
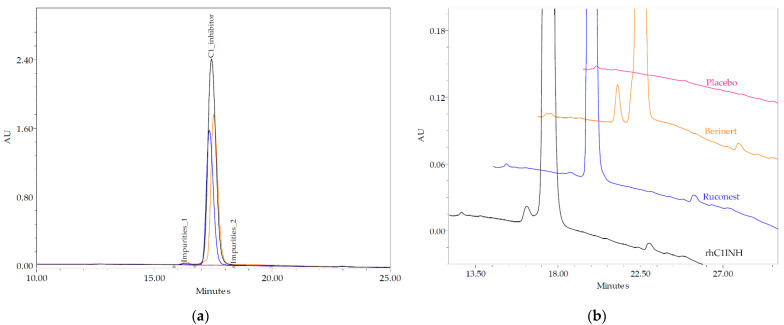
Comparison of reverse-phase chromatograms of the novel rhC1INH (black line), Berinert^®^ (yellow line), Ruconest^®^ (blue line) and placebo (red line); (**a**) full view of the chromatogram, (**b**) enlarged view of impurities peaks.

The reverse-phase chromatography (RP-HPLC) method allows the separation and quantification of hydrophobic variants of the protein molecules, such as oxidation and chain clipping, due to the denaturing effect of the low pH and high organic solvent. The API of Ruconest^®^ showed the highest level of purity of 99.87%. The purity of the rhC1INH was 98.98%, while Berinert^®^ was characterized by 94.27% of purity (Table 4 and Figure 6).

The level of impurities of minor proteoforms with different hydrophobicity (1 and 2) corresponding to forms of the rhC1INH was less than 1%, same as for Ruconest^®^, whereas Berinert^®^ had the highest level (more than 2.7%) of each impurities.

### 2.7. Charge Heterogeneity

#### Isoelectric Focusing (IEF)

**Figure 7:**
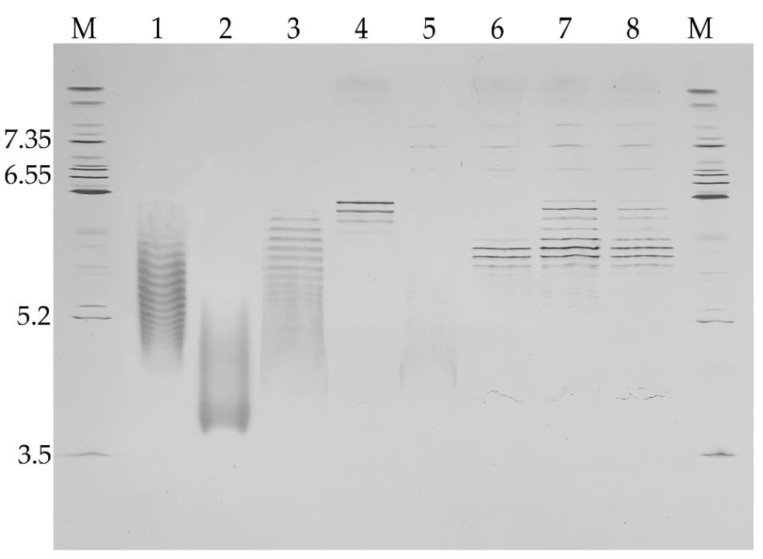
Electropherogram of C1 inhibitor charge variants. #1 Ruconest^®^, #2 Berinert^®^, #3 rhC1INH, #4 O-deglycosylated rhC1INH, #5 N-deglycosylated rhC1INH, #6 total deglycosylated rhC1INH, #7 total deglycosylated Berinert^®^, #8 total deglycosylated Ruconest^®^, M- pI markers.

The C1 inhibitor molecule is heavily glycosylated. As described below, C1INH possesses six occupied N- and more than 12 O- sites of glycosylation, which provide the inhibitory activity [15]. Due to the high content of charged glycans (especially O-type) carrying a high content of sialic acid, the standard methods of IEF (native and denatured) are not suitable for an intact C1 inhibitor, (lanes 1–3 on Figure 7), because of a poor bands’ resolution. 

The comparative IEF analysis of several C1 inhibitor products showed different profiles of charge variants. While Ruconest^®^ has 14 distinct isoforms, Berinert^®^ has no good resolution of its bands. The pI for Berinert^®^ isoforms corresponds to 4.3–5.3 as judged by the intensity distribution of electropherogram (lane2). The rhC1INH has a charge isoform pattern that includes some isoforms present in Berinert^®^ (undetectable part) and most of the isoforms present in Ruconest^®^. The pI range for rhC1INH is 4.5–6.3 and 5.2–6.5 for Ruconest^®^.

To analyze charge isoforms independently of glycosylation type, rhC1INH was subjected to additional enzymatic treatment. No clear bands of rhC1INH isoforms were found for the N-deglycosylated sample with exception of peptide-N4-(N-acetyl-beta-glucosaminyl) asparagine amidase F (PNGase F) isoforms migrating in pH range of 6.9–7.4 (lane 5). Some smear occurred in between 4.8–6.0. The bands’ mobilities of O-deglycosylated and totally deglycosylated rhC1INH were different (lanes 4 and 6), that means O-glycans to affect the total charge of the molecule. Nevertheless, the isoforms resolution becomes better after O-glycans removal.

To compare the novel C1 inhibitor molecule with the commercially available C1INH products, we applied the same enzyme processing to all three samples (Figure 7, lines 6–8) We noticed a similar pattern of major bands distribution within 5.7–6.2 pI range but also detected additional basic isoform’s bands for Berinert^®^ and Ruconest^®^. This could potentially result from additional polypeptide chain modifications or incomplete enzymatic digestion of the samples. The full data of isoforms are present in Table S2.

### 2.8. Glycosylation

**Figure 8:**
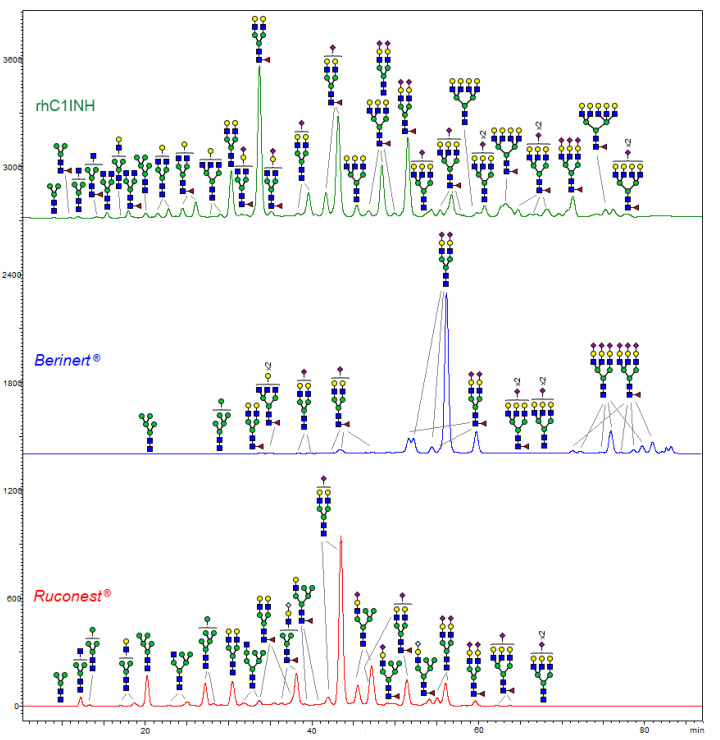
Comparative N-glycan profiles of rhC1INH, Berinert^®^ and Ruconest^®^.

N-glycan analysis of the novel rhC1INH allowed detection of a high degree of heterogeneity compared to the N-glycan patterns of either Berinert^®^ or Ruconest^®^ (Figure 8).

Each N-glycan profile typically demonstrates one major peak accompanied by some smaller species. Berinert^®^ possess a profile with ~60–80% biantennary, disialylated, non-fucosylated (A2G2S2) structures with human-like alpha-2,6-linked sialic acids, which are not essential for biological activity but enhance in vivo half-life and in vitro protein stability [23]. The whole pattern of this plasma-derived product is represented by completely galactosylated (95%) glycans at most biantennary (80%). Polyantennary (tri- or tetra-) species make up a part below 20% [15,24]. Less mature types such as high-mannose and hybrid glycans are present in relatively minor amounts. Together with alpha-2,6-linked sialic acids, complex glycans of Berinert^®^ are composed of alpha-2,3-linked residues that enlarge a heterogeneity of individual species as compared to Ruconest^®^ or rhC1INH [25].

The glycosylation pattern of Ruconest^®^ consists of the major abundant glycan A2G2S1 with only alpha-2,6-linked sialic acids. This product also shows some high-mannose, hybrid and complex biantennary variants, although with a lower abundance. As compared to Berinert^®^, high-mannose and hybrid glycans of Ruconest^®^ are detected to a higher extent.

We found that in the rhC1INH molecule, the general set of N-glycans differs from the patterns of Ruconest^®^ and Berinert^®^. Among the area of neutral N-glycans (up to 28 min), the difference mainly relates to the biantennary complex glycan A2G2F peak. Compared to the marketed drugs, this form is dominating through the entire observed spectrum of modifications. High-mannose forms characteristic of Ruconest^®^ are less pronounced in the rhC1INH. On the contrary, the fraction of polyantennary (from 3 to 6 antennas) glycans is significantly higher (Figure 8) in the rhC1INH. As for sialylated species (from 28 to 75 min), the rhC1INH molecule demonstrates a poor similarity to Ruconest^®^ or Berinert^®^ because the CHO cell line is exclusively able to form α2-3 sialylated derivatives only [26]. The vast majority of complex-type N-glycans contain at least one β-galactose residue. This accounts for the possibility of Gal (α1,3)-Gal motif formation. Nevertheless, this carbohydrate antigen was found only in a trace amount (<0.1%). The other potentially immunogenic N-glycolylneuraminic acid residues (Neu5Gc) were not generally observed. Oligosaccharides with core fucosylation are also inherent (Table 5).

Most of the rhC1INH structures contain up to four N-acetylneuraminic acid (Neu5Ac) residues simultaneously that play a key role in the formation of negatively charged protein isoforms.

**Figure 9:**
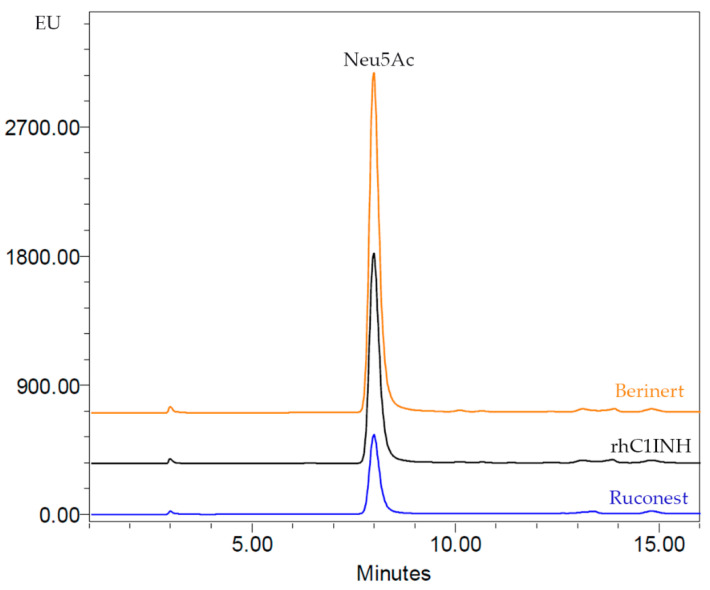
Comparative sialylation profiles of the rhC1INH, Berinert^®^ and Ruconest^®^.

Since N-glycan analysis could mask some minor peaks, we conducted a direct sialylation measurement. The content of Neu5Ac in the protein was determined by reversed-phase HPLC of 1,2-diamino-4,5-methylenedioxybenzene labeled samples. Chromatographic profiles of C1 inhibitor commercial products confirm the presence of only Neu5Ac but not Neu5Gc. The highest content of Neu5Ac was detected for Berinert^®^, while CHO-derived rhC1INH demonstrates a medial value (20 mol sialic acid/mol protein), as compared to the other products (Table 6 and Figure 9). The lowest amount of Neu5Ac was determined for Ruconest^®^.

### 2.9. Biological Activity

We also measured the residual activity inhibition of the C1s subunit, factor XIa, factor XIIa or kallikrein by the rhC1INH with a corresponding chromogenic substrates. The inhibitory activity of the rhC1INH toward various serine proteases was compared to Berinert^®^ and Ruconest^®^. rhC1INH and Ruconest^®^ showed similar activity toward C1s substrate (9.5 and 10 U/mg, respectively calculated in relation to international standard). Berinert^®^ has the lowest biological activity than that of the rhC1INH and Ruconest^®^ (Table 7 and Table 8). As discussed in previous studies [22], a decrease in the purity of C1INH may lead to a decrease in activity. The obtained activity data correlate with SEC results.

**Figure 10:**
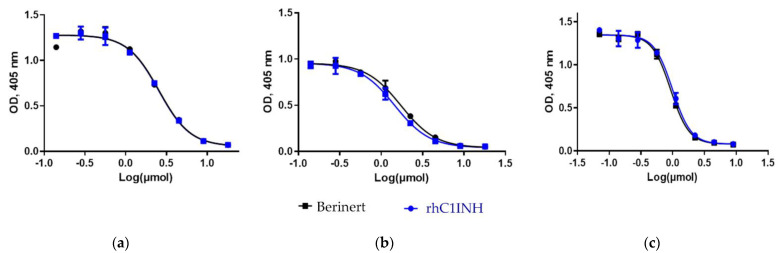
Dose-dependent inhibition of serine proteases by the rhC1INH and Berinert^®^ (chromogenic method): (**a**) blood coagulation factor XIa (FXIa); (**b**) blood coagulation factor XIIa (FXIIa); (**c**) kallikrein.

The study of inhibitory activity toward other proteases (factors XIa and XIIa, and kallikrein, Table 8, Figure 10) showed the similarity between the rhC1INH and Berinert^®^, whose activity was used as a reference (100%). In this research the Ruconest^®^ activity was not analyzed. The recent study of Ruconest^®^ demonstrated the similar biological activity to Berinert^®^ [27]. These differences in the activity between recombinant and plasma-derived C1 inhibitors can be explained by the lower purity of the latter.

Due to the irreversible mechanism of C1 inhibitor-protease complex formation, we calculated only the association rate constant. We determined that rhC1INH produced in CHO cells can form stable complexes with all human proteases analyzed (C1s, FXIa, FXIIa and kallikrein). The association rate constants for rhC1INH purified from CHO were near-to-equal from those for C1 inhibitor purified from human plasma or transgenic rabbit’s milk. Experimental data for rhC1INH are shown in Table 9, while association rate constants for C1 inhibitors purified from other sources were taken from [27].

In addition, a comparative assessment of the anticomplementary activity of the rhC1INH and Ruconest^®^ was determined. The inhibition of human complement by Ruconest^®^ or rhC1INH was evaluated by in vitro study. Samples of human serum were used as a source of complement system components. The complement activity was measured by the lysis of native chicken erythrocytes. Dilution series of Ruconest^®^ (0.0076 to 15.6 IU/mL) or rhC1INH (0.0099 to 20.25 IU/mL) were tested in duplicates for its activity evaluation. The concentrations that inhibits 50% of the complement activity (IC_50_) was calculated from sigmoid regression curves using GraphPad prism 6.0. (Figure S3).

The specific activity of the novel rhC1INH against human C1-esterase in the blood serum was shown to be 1.85 ± 0.49 IU/mL, while it corresponded to 1.66 ± 0.79 IU/mL in case of Ruconest^®^. This indicates similar inhibitory properties against human C1-esterase.

## 3. Discussion

In this study, we assessed product quality attributes of the novel rhC1INH expressed in CHO cells. The extinction coefficient of the rhC1INH is 0.53 (mg/mL)^−1^ × cm^−1^, which is distinct from Berinert^®^ (0.382 mg/mL × cm^−1^) [10] and Ruconest^®^ (0.4 mg/mL × cm^−1^) [27,28]. It means that the rhC1INH has a slightly different conformation influenced by many factors (e.g., glycan profile). We suppose that aromatic amino acids have another accessibility to light absorption.

The molecular mass of total deglycosylated rhC1INH (52,846 kDa) is similar to those previously reported for recombinant and plasma-derived C1 inhibitors [29] what matches with the theoretical value obtained by the amino acid sequence [14]. The detection of the total molecular mass of intact rhC1INH by LC-MS was obstructed by its heavy glycosylation pattern. The number of C1 inhibitor proteoforms with similar molecular weights was too large to achieve well-resolved mass spectra in native mass spectrometry.

Chromato-mass spectrometric interpretation of the primary structure of rhC1INH peaks confirmed 98.6% of the amino acid sequence. Three visible peaks (19, 20 and 21) corresponds to peptides containing glycosylated Asn231, which is involved in the formation and stabilization of the central β-sheet participating in inhibition process. Additionally, we showed that peptide containing Lys307 residue, stabilizing the active center elutes in the peak 14. Peak 26 contains peptides with Ala 443 and Arg444 residues playing a key role in substrate recognition [16,30]. The amino acids involved in the disulfide formation (Cys183 and Cys406) were confirmed for peaks 9 and 26, respectively. However, protein identity cannot be determined up to 100% as region 81–121 contains multiple O-glycosylated sites preventing the protein from proteolytic degradation [15]. We have not analyzed O-glycosylation pattern yet. However, as described previously [25,30,31], the O-deglycosylation affects the ability of C1 inhibitor to form a complex with kallikrein as well as in the case of a truncated type of C1 inhibitor on the surface of endothelial cells. To control O-glycosylation, we used charge distribution IEF analysis. 

We compared the molecular mass of C1 inhibitor fragments by SDS-PAGE and found that under non-reducing conditions, rhC1INH migrates as a 105 kDa monomer likewise pd-C1INH Berinert^®^. Conversely, the molecular weight of recombinant Ruconest^®^ was 98 kDa. The same differences in migration patterns were observed under reducing conditions. Additional impurity bands were identified as glycoforms (103–87 kDa) and fragments (about 30 kDa) previously reported for C1 inhibitor [23]. The observed difference in molecular weight of rhC1INH and Berinert^®^ or Ruconest^®^ accounts for different glycosylation profiles since glycans constitute about 60% of the total protein mass [30].

Being C1 inhibitor a heavily glycosylated protein its glycosylation pattern significantly influences proteolytic specificity, activity and potency [7,9,13].The diversity of N-glycan structures contributes significantly to the heterogeneity of proteins derived from CHO cells. It was shown that N-glycans can potentially affect protein folding, immune regulation, cellular homeostasis, potency, efficiency and the biological half-life of proteins [32]. They are considered a critical quality attribute, and a lot of effort has been put forth to improve the features of protein N-glycosylation. The N-glycans contribute to the conformational stability and kinetics of protein binding to the target proteases. Asn231 and the highly glycosylated N-terminal domain of the C1 inhibitor play an important role in binding to the substrate [9,16]. Previous studies have shown that completely deglycosylated C1INH retains the ability to form a complex with C1s [9,31,33].

A recent study has shown that the high degree of sialylation can prolong the serum half-life of a protein, while desialylation of the protein results in faster clearance from blood, probably because of the affinity of penultimate galactosyl residues to the asialoglycoprotein receptor in the liver [34]. Furthermore, increased rhC1INH sialylation might lead to altered charge distribution, which, as we have shown, can lead to changes in the profile of charge variants on the IEF gel. We detected α2,3-linked sialylated bi-, tri- and tetraantennary glycans in the case of rhC1INH. The sialic acid content of the rhC1INH molecule is in between the range of Ruconest^®^ and Berinert^®^. Thus, we expect that rhC1INH from CHO cells will be cleared from the blood stream faster than Berinert^®^ but significantly slower than Ruconest^®^ (2.4–2.7 h) [35].

In the present study, we assessed the isoform distribution by IEF. We showed that the rhC1INH has a complex charge distribution of basic variants similar to Ruconest^®^ and acidic variants similar to Berinert^®^. Rh-C1INH is characterized by 14 bands within 5.2–6.4 pI and inseparable smear explained by the influence of high sialic acids content of the N-terminal domain, which has been described to be a “rod-like” domain [16]. O-deglycosylation helped to obtain a smaller number of bands and get rid of the inseparable area, while N-deglycosylation did not have positive effect on the acidic variant’s resolution. The total deglycosylated rhC1INH is characterized by seven bands with 5.6–6.2 pI, including three major bands. These data confirmed the idea of sialic acid and O-glycans influence on isoform’s resolution.

The purity of the novel C1INH was assessed by size-exclusion and reverse phase HPLC. The purity of the thC1INH monomer seemed to be as 98.71% that is close to Ruconest^®^ (98.96%) and exceeds respective attributes for Berinert^®^ (74.05%), Cinryze^®^ (69.6%) and Cetor^®^ (67.4%) [22]. Fragments fraction showed the lowest abundance in case of rhC1INH. Based on the RP-HPLC data, the rhC1INH purity corresponds to 98.98 ± 0.2% that is slightly lower than Ruconest^®^ protein and 4.7% higher than that of Berinert^®^. The data from the reverse-phase HPLC allow us to assess the product-release impurities [13].

To evaluate possible therapeutic applications of the novel rhC1INH from CHO in the prophylaxis and treatment of HAE, we performed its biological characterization in comparison with the marketed glycoprotein from rabbit milk (Ruconest^®^).

We showed that the rhC1INH from CHO cells is comparable to Ruconest^®^ in terms of all the main indicators of inhibitory activity (toward FXI, FXII and kallikrein). The difference in the inhibitory activity of rhC1INH and Ruconest^®^ does not exceed 15% (with higher value of inhibition for rhC1INH), that indicates their similar biological activity within presumable error of the method [36].

Similar inhibitory activity toward human complement components was shown for rhC1INH and Ruconest^®^ (does not exceed 11% calculated for Ruconest^®^). The IC_50_ values for the inhibition of non-sensitized anti-erythrocyte antibodies to chicken erythrocytes hemolysis were 1.85 ± 0.49 IU/mL for rhC1INH and 1.66 ± 0.79 IU/mL for Ruconest^®^.

Based on the C1 inhibitor mode of action, we have tested the ability of the rhC1INH to irreversibly bind key human proteases involved in complement and contact activation pathways. We showed that despite their different glycosylation profiles both plasma-derived Berinert^®^ and the rhC1INH from CHO cells have the same order of magnitude of binding to key complement and contact activation pathways participants (Table 9). This confirms the glycosylation difference to be no matter for the specific binding of associated partners.

## 4. Materials and Methods

### 4.1. Materials and Methods

The rhC1INH expressed in the genetically engineered CHO cells was manufactured at JSC “GENERIUM” (Russian Federation) plasma-derived Berinert^®^ (CSL Behring GmbH, King of Prussia, PA, USA), human recombinant C1 inhibitor Ruconest^®^ purified from the milk of transgenic rabbits (conestat alfa, Pharming Group N.V., CR Leiden, The Netherlands).

### 4.2. Extinction Coefficient and Quantitative Content Determination of Protein

The extinction coefficient was determined using the Edelhoch parameter with a Varian Cary 100 spectrophotometer (Agilent Technologies, Santa Clara, CA, USA). The measurement of the absorption of the diluted protein (concentration of about 1.0 mg/mL) was carried out under native (0.2 M Sodium Phosphate pH 6.8) and denaturing (6 M Guanidine-HCl in 0.2 M Sodium Phosphate pH 6.8) conditions as previously described [12,37]. The theoretical molar extinction coefficient for denatured protein was calculated according to [14]. The concentration was determined by measuring the difference in absorption at wavelengths of 280 and 320 nm.

### 4.3. Molecular Mass Determination by LC-ESI-MS

The human C1INH is highly glycosylated and bonded with 6 N-glycans and up to 16 O-glycans. Therefore, a complete deglycosylation step was necessary to analyze the molecular mass distribution in the samples, as previously described in [29].

After replacing the formulation buffer with the reaction buffer containing 500 µg of protein, 10 µL of each sialidase solution from SialEXO (cat. # G1-SM1-020 Genovis, Lund, Sweden) and OglyZOR (cat.# G1-OG1-020 Genovis, Lund, Sweden) and a PNGase F (cat.# P7367-300UN, Sigma Aldrich, St. Louis, MO, USA) solution up to the final concentration of 100 U/mL were added to the samples. Samples were incubated at 37 °C overnight. The reaction was stopped by adding 1% formic acid.

Deglycosylated samples were analyzed by LC-ESI-MS using Nexera X2 HPLC system (Shimadzu Corp., Kyoto, Japan) equipped with a Bioresolve RP mAb Polyphenyl column, 450 Å, 2.7 µm, 1.0 × 150 mm (P/N 176004167, Waters, Milford, MA, USA), and a 6550 QTOF mass spectrometer (Agilent Technologies, Santa Clara, CA, USA). Elution was achieved by a linear gradient of 20–60% of mobile phase B (mobile phase A, 0.15% difluoroacetic acid in water; mobile phase B, 0.15% difluoroacetic acid in acetonitrile) at a flow rate of 0.1 mL/min for 10 min. The range of registered *m*/*z* values in frontal scanning was 700–7000 Th. To process the mass spectra, MassHunter Qualitative Analysis B.08.00 and UniDec v. 4.1.2. [38] (Agilent Technologies, Santa Clara, CA, USA) were used.

### 4.4. Amino Acid Sequence Validation

The method of sequence validation and peptide mapping sample preparation has been previously described [39]. Each sample was diluted to 1.0 mg/mL with denaturing buffer (6 M Guanidine-HCl, 1 mM EDTA, 0.1 M Tris-HCl, pH 7.8). 1 M dithiothreitol (DTT) was added to the final concentration of 5 mmol/L, and samples were incubated at 4 °C for 1 h. Alkylation was performed by adding 0.5 M iodoacetamide (IAM) to the final concentration of 10 mmol/L and incubating at 4 °C for 1 h in the dark. Then the denaturing, reducing and alkylating reagents were removed with a reaction buffer (1 M Urea, 0.1 M Tris-HCl, pH 7.8) using Zeba^TM^ Spin 7K MWCO size-exclusion desalting columns (P/N 89882, Thermo Scientific, Waltham, MA, USA) according to the manufacturer’s instructions.

After this step, the samples were divided into five aliquots of 20 µL. Then about 0.2 µg of chymotrypsin was added to the al. “A”, 0.278 µg trypsin to the al. “B”, 0.2 µg of rAsp-N to the al. “C”, 0.4 µg of Glu-C to the al. “D” and 0.2 µg of Lys-N to the al. “E”. The aliquots “A” and “B” were incubated at room temperature for 4 h, and aliquots “C”, “D” and “E” were at room temperature for 36 h. The digestion was quenched by adding 0.1 µL of formic acid. The experimental data was processed with the “Sequence Validation” algorithm by PEAKS AB software (Bioinformatics Solutions, Waterloo, ON, Canada).

The method of peptide mapping sample preparation is the same as described below (part 4.4) and includes a step of removing a reaction buffer (1 M Urea, 0.1 M Tris-HCl, pH 7.8) using Zeba^TM^ Spin 7K MWCO size-exclusion desalting columns (P/N 89882, Thermo Scientific, Waltham, MA, USA) according to the manufacturer’s instructions.

The analysis was performed in a linear gradient nano-HPLC reverse-phase Acclaim™ PepMap™ 100 C18 LC column, 3 µm, 150 mm length, 0.075 mm I.D. (Thermo Scientific, Waltham, MA, USA) with a mass spectrometry data-dependent acquisition using Orbitrap Q Exactive (Thermo Scientific, Waltham, MA, USA) with HCD fragmentation. The data was processed using Peaks AB v 2.0 (Bioinformatics Solutions, Waterloo, ON, Canada) and BiopharmaFinder v.3.2 (Thermo Scientific, Waltham, MA, USA).

### 4.5. Peptide Mapping

Trypsinolysis for the peptide mapping protocol was performed by adding 5 µg of the enzyme (V5111, Promega, Madison, WI, USA) to the 1000 µg of reduced protein sample with subsequent incubation at 37 °C for 4 h. The reaction was stopped with 1% (*v*/*v*) of TFA addition.

After trypsin digestion, the peptides were separated by reverse-phase HPLC using the Advance Peptide Map C18 column (cat. # 653750-902, 2.7 mm, 2.1 mm × 150 mm, Agilent Technologies, Santa Clara, CA, USA) at 60 °C with tandem high-resolution MS detection. Elution of peptides was achieved by a linear gradient 0–59% of mobile phase B in 60 min (mobile phase A, 0.1% formic acid in 20 mM of Ammonium Formate; mobile phase B, 0.1% formic acid in acetonitrile), at a flow rate of 0.3 mL/min. Peptides were analyzed by the Orbitrap Q Exactive (Thermo Scientific, Waltham, MA, USA) in data-dependent acquisition mode with HCD fragmentation. Data was processed by BiopharmaFinder v.3.2 (Thermo Scientific, Waltham, MA, USA).

### 4.6. High Order Structure

Samples were dialyzed against SP buffer (20 mM sodium phosphate, 50 mM sodium chloride, pH 6.8) in dialysis bags with a 3.5 kDa cut-off limit one time for 15 h and three times for 2 h at 4 °C.

*Differential Scanning Calorimetry (DSC)* was performed using a DASM-4M scanning microcalorimeter (Biopribor, Pushchino, Russian Federation) at the temperature from 20 °C to 90 °C at a rate of 0.86 K/min with overpressure in the cells of 3 bar. The concentration of the sample was 2.0–2.1 mg/mL. The solution was degassed for 15 min under vacuum at 0.7 atm and temperature of 25 °C with constant stirring. The SP buffer was loaded into the measuring cell and the comparison cell, the heating-cooling cycle was established, after which the reproducibility of the calorimetric curve within 1 μW was achieved by several successive reloads of the buffer in the measuring cell.

*Circular Dichroism (CD).* This analytical method measures the difference in left and right-handed circularly polarized light. CD experiments were performed at 20 °C on a spectropolarimeter J-810 (JASCO Corp, Fuji, Shizuoka, Japan), with cells having 1 cm path length for near-UV CD spectra and cells having 0.1 cm path length for far-UV CD spectra. Near-UV CD spectra were recorded from 250 to 350 nm wavelength with step size and bandwidth of 1 nm each; sample concentration was 23.8–24.96 µM. Far-UV CD spectra were recorded in the range of 190–250 nm with the same wavelength step size and bandwidth step size of 0.7 nm. The sample concentration was 0.96–0.99 µM. Far-UV CD spectra were used to measure the secondary structure of proteins, whereas near-UV CD spectra were used to measure the tertiary structure of proteins. Three scans were performed for each sample, and baseline correction was applied. Noise reduction and normalization of the spectrum were performed by use of Spectra Analysis v.1.53.04 (JASCO Corp, Fuji, Shizuoka, Japan) and OriginPro 9.0 (OriginLab Corp., Northampton, MA, USA) software, respectively.

*IR spectra* were measured using a Nicolet 6700 FT-IR (Thermo Scientific, Waltham, MA, USA) spectrometer in transmission mode in a crystalline calcium fluoride cuvette with an optical path length of 4 μm using MCT (Hg, Cd, Te), scanning in the wave range from 650 cm^−1^ to 4000 cm^−1^ with a resolution of 1 cm^−1^, averaging over 256 spectra. IR spectra were measured at 20 °C. The protein concentration was about 60.9 mg/mL. The optical path of the cuvette was (4.52 ± 0.04) μm.

*Intrinsic fluorescence analysis* was performed using a Cary Eclipse fluorescence spectrometer (Agilent Technologies, Santa Clara, CA, USA) at the wavelength range between 290 nm and 420 nm with a step of 2 nm and a data averaging time of 2 s. at each point. The widths of the spectral slit of the analyzing monochromators were 5 nm and 2.5 nm, respectively. The voltage across the photomultiplier tube was 700 V. The protein concentration was 2.0–2.1 µM.

*Ellman’s assay.* The procedure is based on the reaction of the thiol with Ellman’s reagent 5,5′-dithiobis(2-nitrobenzoic acid) (DTNB) to give rise to the mixed disulfide and 2-nitro5-thiobenzoic acid (TNB), which is then quantified by the absorbance of the anion (TNB2−) at 412 nm. Disulfide-linked peptide peaks were detected under non-reducing conditions, and those peaks containing cysteine residues were detected after treatment with dithiothreitol for the reduction in disulfide bonds. The absorbance of each disulfide-linked peptide was analyzed and manually assigned. The amount of free sulfhydryl in the 1 mg/mL of protein was calculated, using the standard value Δε412 = 13,600 M^−1^ cm^−1^ [40].

*Dynamic light scattering measurements*. The signal accumulation of the helium-neon laser was carried out at a wavelength of 632.8 nm, at 25 °C, and a scattering angle of 173°. The accumulation time of the autocorrelation function for one measurement was 200 s. To determine the diffusion coefficients of the solution particles, the refractive index *n_D_* and the dynamic viscosity η of buffer A were measured: *n_D_* = 1.3352 and η = 0.92 mPa·s at 25 ° C. The automatically preset values *n_D_* for the protein were used: refractive index *n_D_* = 1.450, absorption A = 0.01. To estimate the molecular weight of the protein according to the Mark–Houwink equation, empirical parameters K = 7.67 × 10^−5^ cm^2^·s^−1^, a = 0.428 were used.

### 4.7. Size Exclusion Chromatography

Protein was analyzed by TSKgel G3000SWXL column (cat. #00854, 5 μm, 7.8 × 300 mm Tosoh Bioscience, Tokyo, Japan) at a flow rate of 0.5 mL/min with a mobile phase-eluent containing phosphate buffered saline (PBS), with the addition of acetonitrile to 5%, pH 7.4. Samples were detected by UV at 214 nm using HPLC system Alliance e2695/2489 (Waters, Milford, MA, USA). Data were acquired and processed by Empower^TM^ 3 (Waters, Milford, MA, USA) software. Results are presented as the relative monomer, fragments and HMW content.

### 4.8. Reverse-Phase Chromatography

Hydrophobic impurities were measured using YMC-Pack PROTEIN-RP (cat. #PR99S051504QT, 5 μm, 200Å, 4.0 × 150 mm YMC CO., Kyoto, Japan) at a flow rate of 0.5 mL/min with a mobile phase A of 0.1% TFA in HPLC grade water, and phase B of 0.1% TFA in acetonitrile. The linear gradient 5–100% phase B in 30 min was applied. Samples by UV were detected at 214 nm using HPLC system Alliance e2695/2489 (Waters, Milford, MA, USA). Data were acquired and processed by Empower^TM^ 3 (Waters, Milford, MA, USA) software. Results are presented as the relative monomer content and impurities.

### 4.9. Electrophoretic Analysis

SDS-PAGE was performed in non-reducing and reducing (with 50 mM DTT) conditions on Mini-PROTEAN^®^ TGX™ Precast Protein 4–15% Tris-Glycine mini gels (cat. #4561083, BioRad, Des Plaines, IL, USA). Isoelectric focusing (IEF) was performed on IEF CleanGel (S/N 1025641, GE Healthcare, North Richland Hills, TX, USA) per the manufacturer’s instructions.

### 4.10. N-Glycan Analysis

Sample preparation was performed by GlycoPrep^®^ Rapid N-Glycan Preparation with InstantPC^TM^ kit (cat #GP96NG-LB, Prozyme, Pl Hayward, CA, USA). Separation of labeled oligosaccharides was carried out by hydrophilic interaction chromatography using the Shimadzu Nexera X2 HPLC system with RF-20A xs fluorescence detector (Shimadzu Corp., Kyoto, Japan) with AdvanceBio Glycan Map column (2.1 × 150 mm, 120 Å, 2.7 μm, cat. # 683775-913, Agilent Technologies, Santa Clara, CA, USA) at a flow rate of 0.4 mL/min and an elution gradient of 80 mM ammonium formate in water (mobile phase A) with acetonitrile (mobile phase B). A linear gradient was applied: 0 min—75%B, 80 min—60%B, 82 min—40%B, 86.5 min—40%B, 88.5 min—75%B, 96.5 min—75%. N-glycans were detected by the fluorescence signal at 345 nm after 285 nm light excitation and identified by QTOF 6550 (Agilent Technologies, Santa Clara, CA, USA) time-of-flight quadrupole mass spectrometer with a Dual Jet Stream ion source in positive ionization mode within 400–1700 *m*/*z* scanning range. An oligosaccharides search was performed using the MassHunter Qualitative Analysis v.B.07.00 SP2 software with the BioConfirm B.08.00 module (Agilent Technologies, Santa Clara, CA, USA) followed by registered masses filtration using GlycoWorkbench (ver. 2.1) software [41].

### 4.11. Sialic Acid Analysis

The determination of the total sialic acids content was carried out by RP-HPLC. Samples with a concentration of 0.5 mg/mL were subjected to hydrolysis with 2M acetic acid at 80 °C for 2 h. Labeling of the cleaved sialic acids was carried out by incubating samples at 50 °C for 3 h with a fluorescent dye DMB. The analysis of the samples preliminarily diluted 10 times with the mobile phase (9% acetonitrile, 7% methanol in water) was carried out on a Symmetry C18 column (150 × 3.9 mm, 5 μm particles, 100 Å, cat. #WAT046980, Waters, Milford, MA, USA) λ_ex_ = 373 nm, λ_em_ = 448 nm at a flow rate of 0.5 mL/min for no less than 45 min by isocratic mode. Samples were analyzed using the HPLC system Alliance e2695/2475 (Waters, Milford, MA, USA). Data were acquired and processed by Empower^TM^ 3 (Waters, Milford, MA, USA) software.

### 4.12. Biological Activity

A comparison of the specificity and specific activity of the rhC1INH and Berinert^®^ to C1s subunit was evaluated by the chromogenic method according to the manufacturer’s instructions for the TECHNOCHROM^®^ C1-INH Kit (cat. #5345003, Technoclone GmbH, Wien, Austria).

A comparison of the biological activity of the international standard (NIBSC, cat. #08/256), rhC1INH and Berinert^®^ in relation to blood coagulation factors FXIa, FXIIa and human kallikrein was assessed by the chromogenic method. Diluted samples were added to the wells of a non-absorbent plate at the following concentrations: 18 to 0.14 µM for FXIa and FXIIa -, 9 to 0.07 µM for kallikrein. The titration step was performed in 2 rounds. Then first reagent 44 μM FXIa (cat. #HFXIa 1111a, Enzyme Research Labs Inc., South Bend, IN, USA), 44 μM FXIIa (cat. #7691-250, BioVision, Zürich, Switzerland), or 80 μM kallikrein (cat. #K2638, Sigma Aldrich, St. Louis, MO, USA) was added, diluted by 200, 30 and 70 times with a buffer (50 mM Tris and 361 mM Sodium chloride), respectively, followed by incubation at 37 °C for 5 min. Then a chromogenic substrate was added: S-2366 (pyroGlu-Pro-Arg-pNA, cat. #S821090, Ghromogenix, Mölndal, Sweden) for FXIa and kallikrein and S-2302 (HD-Pro-Phe-Arg-pNA, cat. #S820340, Ghromogenix, Mölndal, Sweden) for FXIIa. The volume ratio of the components was 1/1/1. Incubation was performed at 37 °C for 3 min. The reaction was stopped by adding acetic acid. Optical density was measured at 405 nm by Spectra Max M3 (Molecular Devices, San Jose, CA, USA).

### 4.13. C1 Inhibitor Association Rate Constants Determination for Human C1s, FXIa, FXIIa and Kallikrein

Association rate constants were measured using biolayer interferometry on the Octet QKe (ForteBio, Menlo Park, CA, USA). Each protease was diluted to 10 μg/mL in 10 mM sodium acetate (pH 6.0) and covalently immobilized onto 3 amino reactive sensors (AR2G) for each protease achieving the following levels: 0.62 ± 0.05 nm for C1s, 4.0 ± 0.05 nm for FXIa, 2.8 ± 0.10 nm for FXIIa and 2.7 ± 0.25 nm for kallikrein. The additional sensor was used for baseline drift correction. Three non-zero concentrations of C1 inhibitor (11 μM; 5.5 μM; 2.8 μM) were used in triplicates to calculate the association rate constant, and 0 μM was used as a negative control. All measurements were performed at 24 °C at 250 rpm of the internal orbital shaker.

### 4.14. Complement Activity in Human Serum

The dilution samples of the rhC1INH from CHO cells (0.0099 to 20.25 IU/mL) and of Ruconest^®^ (0.0076 to 15.6 IU/mL) were pre-incubated with human serum (1:10 dilution) in the presence of 10% chicken erythrocyte suspension for 1 h at 37 °C. After incubation, chicken red blood cells were removed by centrifugation, and the supernatant was transferred to a new plate and measured by a spectrophotometer at a 415 nm wavelength. The IC_50_ value (concentration that inhibits 50% of the complement activity) was calculated using GraphPad Prism 6.0 (GraphPad Software, Inc., Fay Ave La Jolla, CA, USA).

## 5. Conclusions

This study describes the physicochemical and biological characterization of the novel rhC1INH from CHO cells. The product quality attributes of the rhC1INH were determined by employing state-of-the-art analytical methods.

The extinction coefficient of the rhC1INH was determined by the Edelhoch method and shown to be 0.53 (mg/mL)^−1^ × cm^−1^, which is similar to Berinert^®^ [10] and Ruconest^®^ [27,28].

The molecular mass of totally deglycosylated rhC1INH was determined by LC-ESI-MS and shown to be 52,846 Da. This value corresponds to the previously reported molecular mass of totally deglycosylated plasma-derived C1 inhibitor Berinert^®^ [29].

The primary structure of the rhC1INH was determined by peptide mapping using the RP-HPLC method with almost full sequence coverage (98.6%). The high order structures of the rhC1INH were analyzed by a variety of analytical methods, and our data conform to the published data.

The purity of C1 esterase inhibitor proteins was determined by HPLC methods to assess amounts of aggregates by SEC, the number product-relative impurities with different hydrophobicity and pI by RP and IEF. The results show CHO-derived rhC1INH has an intermediate monomer purity as compared to Ruconest^®^ and Berinert^®^. The visual analysis of charge forms distribution revealed that novel rhC1INH has basic forms similar to Ruconest^®^, while acidic isoforms profile reminds that of Berinert^®^. These findings were further confirmed by direct N-glycan analysis. We detected a different sialylation that is typical for Berinert^®^ and Ruconest^®^, i.e., α2,3-linked or α2,6-linked sialic acid residues. The rhC1INH molecule has α2,3-linked sialylated species only that accounts for similarity of Berinert^®^ acidic isoforms. A more detailed analysis of sialic acids content was performed via RP-HPLC. The results demonstrate the intermediate amount of Neu5Ac in the rhC1INH form CHO cells as compared to the marketed C1 inhibitors. Thus, we expect that rhC1INH plasma clearance will be faster than that of Berinert^®^ but significantly slower than that of Ruconest^®^ (2.4–2.7 h) [35]. A more detailed analysis of sialic acids content was performed by RP-HPLC. The results demonstrate the intermediate amount of Neu5Ac in the rhC1INH form CHO cells compared to the marketed C1 inhibitors. O-glycosylation patterns have not been analyzed.

We also analyzed the molecular weight and proteoforms heterogeneity by the SDS-PAGE method. It appears that rhC1INH has a molecular mass similar to Berinert^®^, while Ruconest^®^ migrates faster. Despite the identical primary structure, all three C1 esterase inhibitor products demonstrate apparent molecular weight distribution that is a consequence of different glycosylation profiles [30].

Similar inhibitory activities of rhC1INH and Ruconest^®^ were shown by the chromogenic method with substrates such as C1s subunit and human complement components. In a comparative study the difference of the inhibitory activities of rhC1INH, Ruconest^®^ and Berinert^®^ does not exceed 15%. This confirms a high similarity of the biological activity of C1 esterase inhibitor-based therapeutics [36].

As described above, the difference in glycoprotein profile between rhC1INH and pd-C1INH Berinert^®^ does not affect its biological activity and ability to regulate complement, contact, fibrinolytic and coagulation systems.

Based on all the above, one can conclude that novel rhC1INH expressed in CHO cells has physicochemical and biological properties similar to both Ruconest^®^ and Berinert^®^ and thus may be used for the treatment of pathologic conditions caused by C1INH deficiencies, such as HAE. The main advantages of rhC1INH produced in CHO cells are high yields, stability and the functional activity required for therapeutic scale production, and the absence of blood-borne pathogens has been achieved.

We also conclude that the rhC1INH from CHO cells can be further tested preclinically as a possible therapeutic protein for the treatment and prophylaxis of HAE/AAE.

## 6. Patents

Patent # 2651778 and 2663464 “Combinatorial therapy for hemorrhagic shock treatment” until 18/09/2035.

## Figures and Tables

**Table 1:** The LC-MS data on the mass of the deglycosylated rhC1INH.

PTM	Mass, Da	Amount, %
_−1_N	52,732	5.1
_−1_NP_2_	52,636	4.8
_−1_NPN_3_	52,522	12.0
_−1_NPNATSSSSQD_11_	51,754	7.2
rhC1INH (full structure)	52,846	71.0

**Table 2:** The major protein bands identified by SDS-PAGE in the rhC1INH, Ruconest^®^ and Berinert^®^.

Samples	Molecular Weight, kDa			
	Non-Reduced		Reduced	
	Major Band	Minor Band	Major Band	Minor Band
Ruconest^®^	95	86	94	82; 29
Berinert^®^	114	103 90	108	101 92 87 31
rhC1INH	125	109	113	97; 30

**Table 3:** The purity of rhC1INH, Ruconest^®^ and Berinert^®^ measured by SEC.

Sample	Content, %			Retention Time (Main Peak), min
	Aggregates	Main Peak	Fragments	
rhC1INH	0.96	98.71	0.33	14.06
Ruconest^®^	0.45	98.96	0.58	14.73
Berinert^®^	3.09	74.05	22.87	13.98

**Table 4:** The purity estimation of the compared samples by RP-HPLC.

Sample	Content, %			Retention Time (C1 Inhibitor), min
	Impurities 1	C1 Inhibitor	Impurities 2	
rhC1INH	0.76	98.98	0.26	17.42
Ruconest^®^	0.13	99.87	* N/D	17.33
Berinert^®^	2.77	94.27	2.96	17.52

* not detected.

**Table 5:** The relative area (%) of glycan peaks in rhC1INH based on HILIC data.

N-Glycan Groups	Content, %
Charged	55 ± 5
Neutral	45 ± 5
Fucosylated	90 ± 5
Galactosylated	97 ± 2
Complex biantennary	50 ± 10
Complex multiantennary	50 ± 10
High mannose	Less than 1
Neu5Gc	Not detected
Gal(α1,3)-Gal	Less than 0.1

**Table 6:** The Neu5Ac content (mol/mol protein) determined by RP-HPLC.

Samples	Mean ± SD
rhC1INH	20.4 ± 1.1
Ruconest^®^	8.0 ± 0.6
Berinert^®^	35.0 ± 1.0

**Table 7:** The specific/biological activity of C1INH toward C1s subunit.

C1 Inhibitor	Biological Activity, U/mL	Specific Activity, U/mg
rhC1INH	175	9.5
Ruconest^®^	166	10
Berinert^®^	54	8.7

**Table 8:** Inhibition of C1s subunit by C1INH.

Samples	Factor XIa	Factor XIIa	Kallikrein
rhC1INH	103%	115%	112%
Berinert^®^	100%	100%	100%

**Table 9:** Comparison of association with proteases.

C1 Inhibitor	k_on_ (1/Ms)			
	C1s	Factor XIa	Factor XIIa	Kallikrein
rhC1INH	1.67 ± 0.05 × 10^4^	13.8 ± 1.4 × 10^2^	3.26 ± 0.17 × 10^3^	2.86 ± 0.32 × 10^3^
* Ruconest^®^	6.1 ± 0.3 × 10^4^	9.8 ± 0.5 × 10^2^	6.9 ± 0.5 × 10^3^	9.1 ± 0.1 × 10^3^
* Berinert^®^	6.2 ± 0.4 × 10^4^	3.9 ± 0.3 × 10^2^	4.5 ± 0.3 × 10^3^	7.8 ± 0.4 × 10^3^

* data taken from [27].

## Data Availability

Data is contained within the article and supplementary material.

## Supplementary Materials

The following are available online at https://www.mdpi.com/article/10.3390/ph14111180/s1, Table S1: Identified intensity peptide peaks of rhC1INH by LC-MS. Table S2: Isoforms distribution data of C1 inhibitor by IEF. Figure S1: CD spectra (a) in the far UV region and (b) in near UV region; (c) FTIR (1700–1500 cm^−1^) spectra of an α-helix and of a β-sheet protein; (d) Intrinsic fluorescence of the developed rh-C1in. Figure S2: DSC thermograms of rhC1INH. Figure S3: Complement (anti-human C1) specific activity of drugs, estimated by the level of lysis of red blood cells of a chicken. (a)—Ruconest ® from 15.6 IU/mL in steps 2, (b)—rhC1INH from 20.25 IU/mL in steps 2. Final dilutions of human serum 1:10.
