# Supplementary material for: Physicochemical and Biological Characterization of rhC1INH Expressed in CHO Cells
Source: Pharmaceuticals (Basel). 2021 Nov 19;14(11):1180. doi: 10.3390/ph14111180 (PMC8621594; doi:10.3390/ph14111180)
Supplement: Supplementary file 1 [file pharmaceuticals-14-01180-s001.zip › pharmaceuticals-1438688-supplementary.pdf]

Supporting Information Available

Physicochemical and Biological Characterization of rhC1INH Expressed in CHO Cells

Ekaterina Zubareva [1*], Maksim Degterev[1], Alexander Kazarov [1], Maria Zhiliaeva [1], Ksenia Ulyanova [1], Vladimir Simonov [1], Ivan Lyagoskin [1], Maksim Smolov [1], Madina Iskakova [1], Anna Azarova [1], Rahim Shukurov [1]

JSC "GENERIUM", 14, Vladimirskaya Street, Volginskiy 601125, Vladimir Region, Russia zubareva@ibcgenerium.ru, degterev@ibcgenerium.ru, kazarov@ibcgenerium.ru, zhiliaeva@ibcgenerium.ru, ulyanova@ibcgenerium.ru, simonov@ibcgenerium.ru, lyagoskin@ibcgenerium.ru, smolov@ibcgenerium.ru, iskakova@ibcgenerium.ru, azarova@ibcgenerium.ru, shukurov@ibcgenerium.ru.

* Correspondence: zubareva@ibcgenerium.ru. Tel.: +7-(49243)-7-31-04

Table S1. Identified intensity peptide peaks of rhC1INH by LC-MS.

Peak number	Amino acid sequence	Posttranslational modification
1	ISR (252-254)	None
2	GFTTK (190-194)	None
3	NSVIK (295-299)	None
4	VYDPR (473-477)	None
	LEMSK (364-368)	Oxidation
	NSVIK (295-299)	Deamidation
5	LEMSK (364-368)	None
6	TTFDPKK (279-285)	None
7	TTFDPK (279-284)	None
	TLYSSSPR (220-227)	None
	GEGKVATTVISK (19-30)	GalNAc-6GGn-3SG
	DTFVNASR (212-219)	Different type of glycosylation
	VPMMNSK (300-306)	None
8	VATTVISK (23-30)	None
9	DFTCVHQALK (180-189)	Carbamidomethylation
10	LYHAFSAMK (131-139)	None
11	LLDSLPSDTR (255-264)	None
12	MEPFHFK (288-294)	None
13	NSVIKVPMMNSK (295-306)	None
14	KYPVAHFIDQTLK (307-319)	None
15	HRLEDMEQALSPSVFK (343-358)	None
	VTTSQDMLSIMEK (381-393)	None
	YPVAHFIDQTLK (308-319)	None
16	IKVTTSQDMLSIMEK (379-393)	None
17	TNLESILSYPK (169-179)	None
18	LEDMEQALSPSVFK (345-358)	None
19	VLSNNSDANLELINTWVAK (228-246)	Different type of glycosylation
20		
21		
22	FQPTLLTLPR (369-378)	None
23	GVTSVSQIFHSPDLAIR (195-211)	None

24	LVLLNAIYLSAK (265-276)	None
	VGQLQLSHNLSLVILVPQNLK (322-342)	Glycosylation
	MLFVEPILEVSSLPTTNSTTNSATK (31-55)	Glycosylation
25	TLLVFEVQQPFLFVLWDQQHKFPVFMGR (445-472)	None
26	LEFFDFSYDLNLCGLTEDPDLQVSAMQHQTVLELTETGVEAAAASAISVAR (394-444)	Carbamidomethylation
27	KVETNMAFSPFSIASLLTQVLLGAGENTK (140-168)	Oxidation
28	KVETNMAFSPFSIASLLTQVLLGAGENTK(140-168)	None
29	VETNMAFSPFSIASLLTQVLLGAGENTK (141-168)	None

Table S2. Isoforms distribution data of C1 inhibitor by IEF.

Sample	pI	Band, %
Ruconest®	6,45	3,5
	6,37	5,5
	6,28	7,0
	6,17	9,0
	6,00	10,0
	5,91	10,3
	5,78	9,9
	5,67	8,8
	5,59	7,9
	5,52	7,1
	5,47	5,9
	5,38	4,8
	5,31	4,2
	5,22	2,3
	5,15	3,8
Berinert®	5,30	56,5
	4,31	43,5
rhC1INH	6,4	1,4
	6,3	3,0
	6,2	4,4
	6,0	5,1
	5,9	5,5
	5,8	5,9
	5,7	6,2
	5,6	5,2
	5,5	5,4
	5,4	5,6
	5,4	5,0
	5,3	5,2
	5,2	9,6
	5,0	12,3
	4,8	20,2
O-deglycosylated rhC1INH	6,5	36,5
	6,4	30,4
	6,3	6,1
	6,3	18,1
	6,1	6,7
	6,0	2,2
N-deglycosylated rhC1INH	7,4 (PNGase F)	2,7

	7,2 (PNGase F)	4,2
	6,9 (PNGase F)	4,6
	6,0	1,4
	5,9	1,3
	5,8	1,2
	5,7	1,7
	5,5	2,1
	5,5	2,9
	5,4	3,0
	5,3	3,3
	5,2	3,0
	5,2	6,7
	5,0	15,3
	4.8	46.8
total deglycosylated rhC1INH	7,4 (PNGase F)	2,8
	7,2 (PNGase F)	4,1
	6,9 (PNGase F)	2,6
	6,2	2,4
	6,1	7,1
	6,0	25,0
	5,9	30,0
	5,8	16,6
	5,7	7,7
	5,6	1,8
total deglycosylated Berinert®	7,4 (PNGase F)	1,3
	7,2 (PNGase F)	2,4
	6,9 (PNGase F)	1,5
	6,5	2,9
	6,4	10,0
	6,4	5,8
	6,2	5,6
	6,1	11,9
	6,0	22,3
	5,9	18,3
	5,8	8,6
	5,7	3,5
	5,6	1,4
	5,5	2,4
	5,4	1,5
	5,3	0,6
total deglycosylated Ruconest®	7,4 (PNGase F)	1,5
	7,2 (PNGase F)	4,3
	6,9 (PNGase F)	2,7
	6,5	3,2
	6,4	6,5
	6,4	6,3
	6,2	5,4
	6,1	10,3
	6,0	23,1
	5,9	21,5
	5,8	11,5
	5,7	3,7

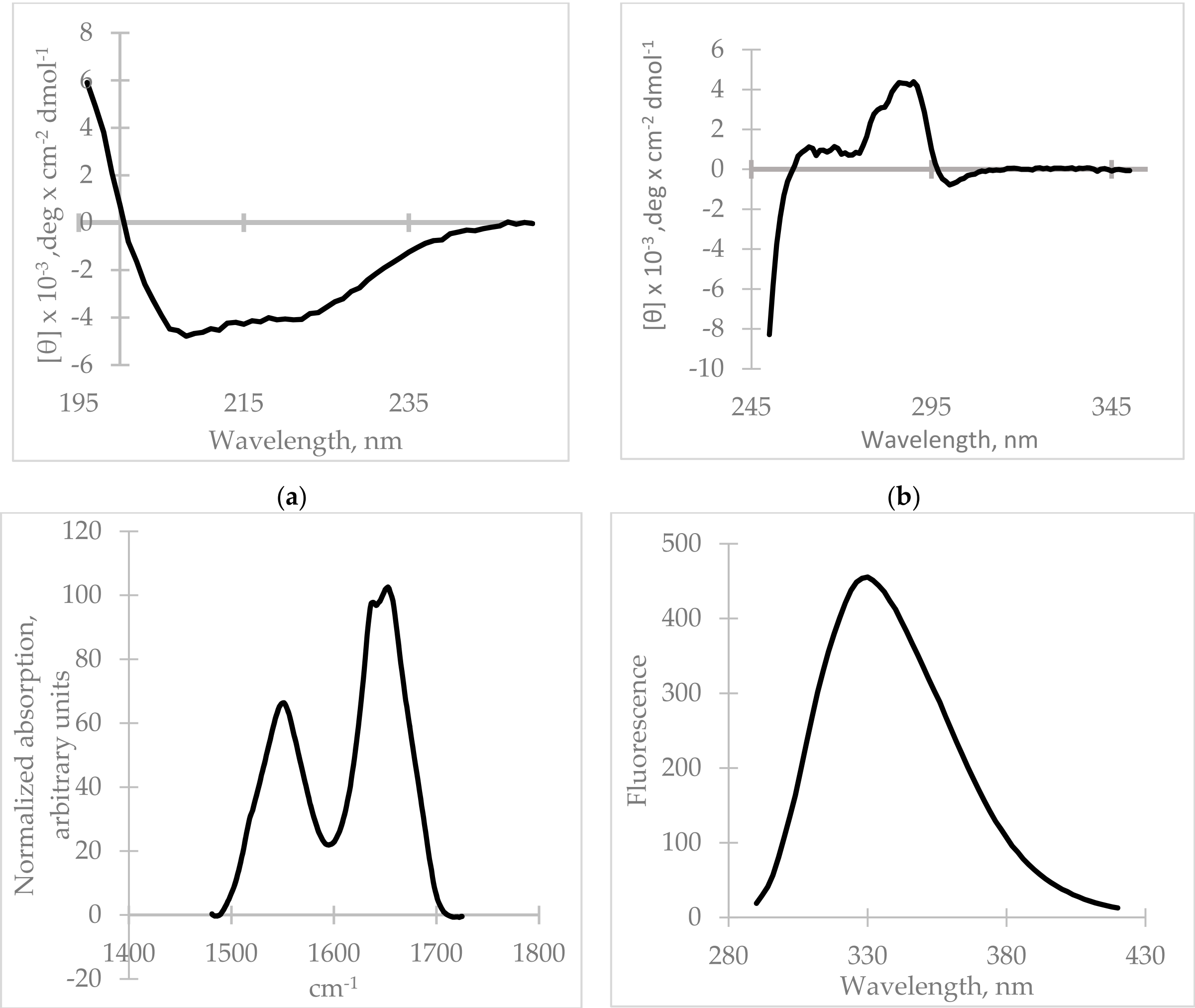

(c) (d)

Figure S1. CD spectra (**a**) in the far UV region and (**b**) in near UV region; (**c**) FTIR (1700–1500 cm⁻¹) spectra of an α-helix and of a β-sheet protein; (**d**) Intrinsic fluorescence of the developed rhC1INH.

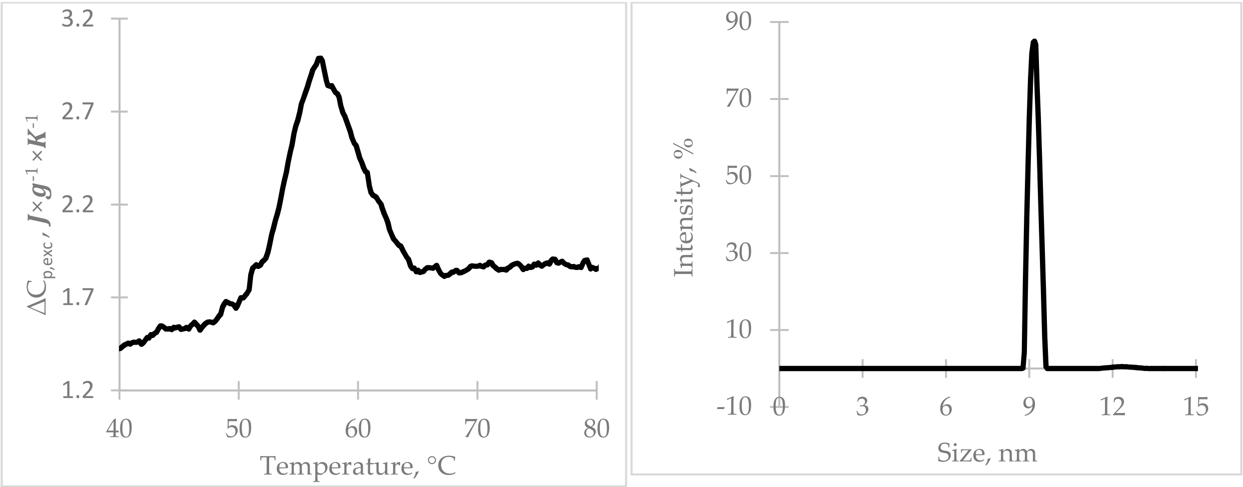

(**a**) (**b**)

Figure S2. DSC thermograms (**a**) and DLS data (**b**) of rhC1INH.

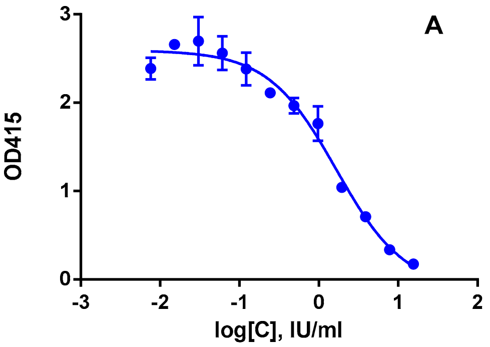

(**a**)

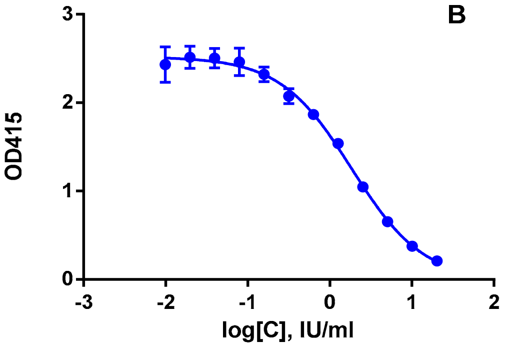

(**b**)

Figure S3. Complement (anti-human C1) specific activity of drugs, estimated by the level of lysis of red blood cells of a chicken. (**a**) - Ruconest® from 15.6 IU/mL in steps 2, (**b**) – rhC1INH from 20.25 IU/mL in steps 2. Final dilutions of human serum 1:10.
